# Paraneoplastic anti-GAD65 extralimbic encephalitis presented with epilepsy: A case report

**DOI:** 10.1097/MD.0000000000034780

**Published:** 2023-11-03

**Authors:** Si-Qi Xia, Hao-Nan Fan, Lin-Feng Fan, Wu Xia, Gao Chen

**Affiliations:** a Department of Neurosurgery, Second Affiliated Hospital, School of Medicine, Zhejiang University, Hangzhou, Zhejiang, China; b Clinical Research Center for Neurological Diseases of Zhejiang Province, Hangzhou, Zhejiang, China; c Department of Respiratory and Critical Care Medicine, Affiliated Hospital of Jiujiang University, Jiujiang, Jiangxi, China; d Department of Neurosurgery, Fourth Affiliated Hospital, School of Medicine, Zhejiang University, Yiwu, Zhejiang, China.

**Keywords:** autoimmune encephalitis, epilepsy, GAD65, paraneoplastic, small cell lung cancer

## Abstract

**Rationale:**

Autoimmunity targeting glutamic acid decarboxylase 65 (GAD65) is associated with type 1 diabetes mellitus as well as various neurological diseases. In the central nervous system, GAD65 autoimmunity usually presents with limbic encephalitis, whereas extralimbic encephalitis (ELE) has only been reported in a few cases. Moreover, anti-GAD65 ELE in the paraneoplastic context has not yet been reported.

**Patient concerns:**

A 60-year-old man presented with intermittent cough and sputum for 10 years, with no other diseases. The patient presented with recurrent seizures that were resistant to antiepileptic drugs (AEDs). Chest computed tomography and pathological results confirmed the diagnosis of small cell lung cancer. Paraneoplastic testing found a high level of GAD65 antibodies in his serum, and cerebrospinal fluid analysis revealed lymphocytic pleocytosis, indicating autoimmune encephalitis. Brain magnetic resonance imaging showed multifocal T2 fluid-attenuated inversion recovery hyperintensities in the extralimbic areas including the subcortex and deep white matter of the bilateral frontal lobe, parietal lobe, and insula lobes.

**Diagnoses:**

Finally, a diagnosis of anti-GAD65 autoimmune ELE with a paraneoplastic etiology from the small cell lung cancer was suspected.

**Interventions:**

The patient refused any tumor-suppressive treatment or immunotherapy for potential side effects and only received AEDs levetiracetam, sodium valproate, and diazepam.

**Outcomes:**

The epilepsy of the patient was resistant to AEDs, and the patient died a week after discharge due to disease progression.

**Lessons:**

Anti-GAD65 autoimmune encephalitis can be extralimbic, can present with isolated epilepsy, and extralimbic anti-GAD65 encephalitis can occur with an underlying malignancy.

## 1. Introduction

Glutamic acid decarboxylase (GAD) 65 is an intracellular antigen highly expressed in the central nervous system (CNS) inhibitory neurons. It is the rate-limiting enzyme in the synthesis of the inhibitory neurotransmitter γ-aminobutyric acid (GABA) and has a significant role in the excitatory-inhibitory balance of the neural circuits. Glutamic acid decarboxylase 65 (GAD65) antibody (Ab)-associated autoimmune encephalitis (AE) usually presents with symptoms of limbic encephalitis (LE) including temporal lobe epilepsy, cognitive deficit, and psychiatric disturbance.^[1]^ It is regarded as typically not related to an underlying tumor, although the risk of cancer increases with age, male sex, and the coexistence of neuronal cell-surface antibodies.^[2]^ GAD Ab-related extralimbic encephalitis (ELE) without limbic symptoms is very rare and has only been reported in 2 cases,^[3,4]^ both presented with isolated epilepsy. What’s more, anti-GAD65 ELE in the paraneoplastic context has not been reported by far. Here, we report a case of GAD65 Ab-associated ELE following small cell lung cancer (SCLC) presented as epilepsy, with no other symptom or temporal lobe abnormality in magnetic resonance imaging (MRI).

## 2. Case report

**Figure 1.**
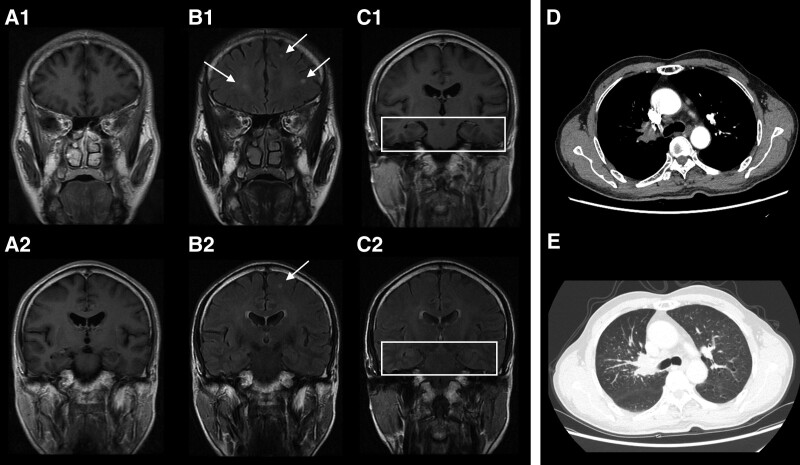
(A–B) T1W and T2W FLAIR imaging showed bilateral multifocal spotty and patchy signals in the subcortex and deep white matter of the bilateral frontal lobe, parietal lobe, and insula lobes. (C) No mesial temporal lobe abnormalities were seen in MRI. (D–E) Enlargement of hilum, amorphous soft tissue density shadow, and multiple small nodules and spot shadows in the right upper lobe of the lung; truncation and occlusion in the bronchus of the right upper lobe of the lung; and multiple enlarged lymph nodes in the mediastinum. FLAIR = fluid-attenuated inversion recovery, MRI = magnetic resonance imaging.

**Figure 2.**
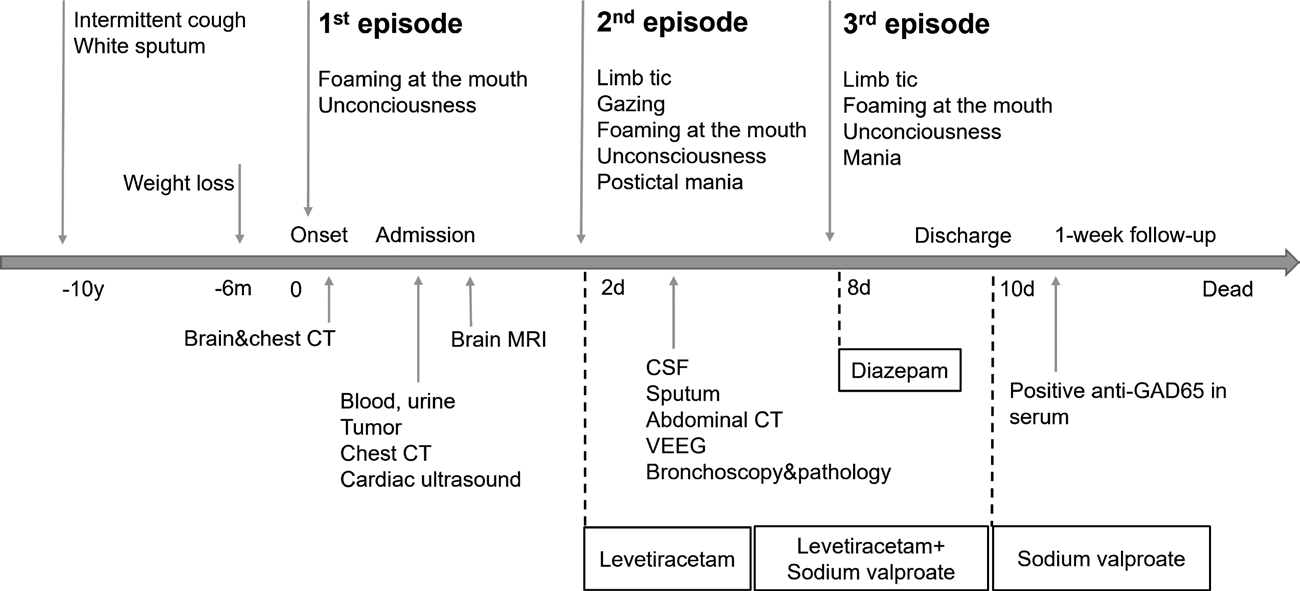
Time course of the patient with data on symptom presentations, diagnostics, and treatments.

A 60-year-old man was admitted to the hospital due to a sudden seizure the last night while sleeping, which presented with foaming at the mouth for several minutes and unconsciousness for nearly 30 minutes. He had an intermittent cough and white sputum for 10 years and otherwise had no medical history. He reported smoking and drinking for 35 years. His family history was unremarkable. Local hospital chest computed tomography (CT) showed a space-occupying lesion in the right lung hilum, but brain CT was normal. Physical examinations including neurological examinations were normal. Blood tests showed decreased lymphocyte percentage (13.9%), and increased levels of tumor markers CEA (12.51 ng/mL) and CYFRA21-1 (7.6). Brain MRI imaging showed bilateral multifocal lesions in the subcortex and deep white matter throughout the frontal lobe, parietal lobe, and insula lobes, which appeared isointense on T1WI and hyperintense on T2W fluid-attenuated inversion recovery sequence. The lesions had no contrast enhancement (Fig. 1), and were rated as Fazekas scale level 2. No mesial temporal lobe abnormality was seen. Chest CT showed nodules and shadows in the right hilum, right upper lobe, and left lower lobe of the lung, which had progressive enhancement; truncation and occlusion in the dorsal bronchus of the right upper lobe and the left lower lobe of the lung; and multiple enlarged lymph nodes in the mediastinum (Fig. 1D and E). On the second night after admission, he suffered the second episode of seizure. It was a generalized tonic-clonic seizure, lasting about 2 minutes, and was characterized by limb tics, foaming at the mouth, bilateral right gaze, unconsciousness, and postictal mania. Levetiracetam treatment (0.5g PO bid) was started, and sodium valproate tablets (0.5g PO bid) were added 4.5 days later. Cerebrospinal fluid (CSF) examination showed pleocytosis (24 cells/μL, mostly lymphocytes), while other CSF results including a series of bacterial and fungal tests were normal. Electroencephalogram (EEG) showed abnormal amplitude modulation of α-waves. Sputum tests showed increased squamous cells, neutrophils, and dust cells. Bronchoscopy found abnormal mucous membrane of the right upper lobe bronchus, which were found positive for CD56, CK7, Ki-67 (+80%), Syn, TTF-1, but negative for CgA, CK5/6, NapsinA, P40, and p63 by subsequent immunohistochemistry. Cytology identified tumor cells in the bronchus tissue. He experienced the third episode at night 1 day after the addition of sodium valproate treatment. It presented first with unconsciousness, foaming at the mouth, limb tics, and then with mania 1 minute later. After that, diazepam (1 ml) was injected intravenously. Paraneoplastic neurological syndrome (PNS)-Ab tests found high titer GAD65 Abs in serum (positive immunoblot assays),^[5]^ while Zic4, Tr (DNER), SOX1, Ma2, Ma1, Amphiphysin, CV2, Ri, Yo, and Hu antibodies were negative. He had no psychiatric or cognitive dysfunction, and neurological examinations were normal. He refused any tumor-suppressive treatment or immunotherapy for potential side effects and was discharged 8 days after admission. After discharge, he only took sodium valproate tablets (0.5g PO bid). He died a week after discharge due to disease progression. The symptoms, diagnostics, and treatments were organized as a timeline (Fig. 2).

## 3. Discussion

This patient had recurrent epilepsy following SCLC, which was resistant to antiepileptic drugs. It should be differentiated from idiopathic epilepsy considering its isolated property. The epilepsy semiology suggested an extralimbic origin, although the limbic system may be temporally affected due to the presence of mania during the seizure episodes. CSF examinations suggested inflammation in the brain and ruled out any bacterial or fungal infection. PNS-Ab screening showed high levels of GAD65 Abs in serum by immunoblot test. Although serum GAD antibodies can be detected in many non-neurological disorders and intrathecal synthesis of GAD Abs needs to be confirmed in most cases, no criteria have been established by far that indicate whether GAD Abs are linked to neurological syndromes.^[5]^ In this patient, the intrathecal synthesis of GAD65 Abs was not examined, but previous literature showed that high GAD65 Ab titers in serum had a high specificity for neurological autoimmunity, and CSF GAD Abs can be detected in 80% of patients with positive serum antibodies.^[6]^ The results lead to the suspected diagnosis of GAD65 Ab-related AE that may result from immunoreactions against SCLC. The lesions in brain MRI of the patient may be due to aging, or may suggest inflammation or demyelination resulting from autoimmune reactions. The mesial temporal lobes were spared from visible MRI damage. Finally, he was diagnosed as GAD65 Ab-associated ELE presented as epilepsy, with a possible paraneoplastic etiology from SCLC.

An epidemiological study found that GAD65 Abs were detected in about 12.5% of unexplained newly-onset epilepsy, and 17% of LE in adults.^[7]^ Patients with GAD65-epilepsy usually have systemic autoimmune disorders, which are mainly type 1 diabetes mellitus and thyroiditis.^[8]^ Also, there might exist concurrent γ-aminobutyric acid (B) receptor-Ab in serum, as well as anti-TPO, antinuclear, antigliadin, and antiphospholipid Abs. The most common type of GAD65-epilepsy is subacute/acute pharmacoresistant temporal lobe epilepsy with LE. Patients usually present with seizures first, but can also have psychiatric, cognitive, or somatosensory dysfunctions. T2W and/or fluid-attenuated inversion recovery MRI typically show mesiotemporal MRI abnormalities indicating LE, which includes bilateral or unilateral mesial temporal hyperintensities, amygdala enlargement, and hippocampal sclerosis or atrophy.^[5]^ The second most common type of GAD65-epilepsy is indolent, slowly-evolving epilepsy without clinical or MRI evidence of active CNS inflammation.^[5,6]^ Other epilepsy semiology that is related to GAD65 include mild non-pharmacoresistant epilepsy, progressive encephalomyelitis with rigidity and myoclonus, palatal myoclonus, myoclonic seizures, musicogenic epilepsy, subtle focal status epilepticus, pilomotor seizures, and idiopathic generalized epilepsy including juvenile myoclonic epilepsy.^[5,9–11]^ In most GAD65-epilepsy patients, the CSF WBC count was normal or mildly elevated. The EEG was usually nonspecific, most likely demonstrating temporal lobe ictal or interictal discharges.^[6]^

The pathogenesis of GAD65 Ab-associated epilepsy has been proposed as decreased presynaptic GABA caused by GAD65 Abs, whether by direct humoral immune response or indirect T-cell-mediated damage.^[6]^ GAD mainly locates in neurons in the CNS, consisting of 2 isoforms GAD65 and GAD67. The GAD65 isoform mainly concentrates in the presynaptic terminal of the inhibitory neuron’s axon, where its N-terminal domain anchors it to synaptic vesicle membranes. It mediates activity-dependent GABA synthesis when postsynaptic inhibition is needed. A decrease of GAD65 in the neurons leads to a decrease of presynaptic GABA by reducing its synthesis and interfering with its exocytosis. The dysfunction of GABAergic inhibitory circuits results in the excitatory-inhibitory imbalance, and contributes to motor function diseases such as epilepsy and stiff-person syndrome (SPS). A study observed significantly decreased basal GABA levels in the brain, as well as enhanced susceptibility to seizures in GAD65 knockout mice.^[12]^ Another study found low cortical GABA levels by magnetic resonance spectroscopy and high serum GAD65 Abs levels in epileptic patients.^[13]^ However, whether GAD65 Abs play a directly pathogenic role or act as biomarkers of T-cell-mediated neuronal damage remains debated, because of the fact that GAD65 is a cytosolic antigen and some contraindicatory experimental results.^[14,15]^ Also, in a patient with GAD65-related LE and seizures, early immunotherapy led to a good outcome and decrease of GAD65 Abs in serum, but GAD65 Abs remained at a very high level during the follow-up, suggesting that the damage was more likely caused by the subsequent immunoreactions than the direct effects of Abs.^[16]^ Studies found that the cluster of differentiation 8 positive (CD8+) T-cells played a major role in the neuronal damage in CNS inflammation through perforin-dependent electrical silencing, which did not necessarily cause neuronal death but could cause chronic epilepsy.^[6]^ This nonlethal cytotoxic damage to GAD65-expressing GABAergic interneurons and ensuing cellular silence/dysfunction can contribute to the formation of seizure foci and epileptic networks, thus lead to epilepsy. In an immunopathology study, the cytotoxic T-cells, GAD65-specific T-cells, and GAD65-specific T cell receptors were observed in GAD65-epilepsy patients, although GAD65 encephalitis generally had milder inflammation and relatively lower CD8/CD3 ratios in the brain tissues compared with the classic intracellular antigen-onconeural cases.^[17]^

With either mechanism, GAD65-epilepsy is associated with widespread inflammation in patients’ brains, which may or may not manifest in brain MRI. A study divided the clinicopathological manifestations of GAD65-epilepsy into 3 stages according to the epilepsy etiology and disease course:^[1]^ (1) reversible ongoing immune activation in the brain with no visible change in MRI; (2) subtle irreversible brain tissue damage manifested in MRI, associated with refractory epilepsy; (3) progressive damage including hippocampal sclerosis and more diffuse brain damage, which form the underlying structural causes of seizures and cognitive symptoms. In terms of brain regions, patients usually first develop hippocampal hyperintensity or even sclerosis in MRI, as the cytotoxic process seems to initially involve limbic areas.^[17]^ Later in the disease course, the pathological change could spread to the extralimbic areas or the contralateral hemisphere, causing progressing signs in brain MRI.^[6]^ A study using diffusion tensor imaging showed that patients with GAD-associated LE had widespread white matter changes across various regions of the brain, suggesting there was more widespread damage in the white matter beyond the limbic system.^[18]^

Clinically, GAD65-associated AE can be extralimbic or limbic. The extralimbic subtype is clinicoradiologically more heterogenous than the limbic subtype, depending on the involved damaging locations. Anti-GAD65 ELE without LE is very rare and has been reported in only 5 cases in adults (Table 1),^[3,4,21,22]^ among which only 2 had no limbic symptoms.^[3,4]^ It is noteworthy that all of the 5 patients were women, and all presented with pharmacoresistant epilepsy. The seizure semiology included simple partial seizure, complex partial seizure, generalized seizure, tonic-clonic seizure, generalized tonic-clonic seizure, status epilepticus, and epilepsia partialis continua. CSF WBC counts were increased in 3 patients; CSF protein levels were increased in 2 patients; and oligoclonal bands were positive in half that had been tested. The brain MRI imaging of all the patients found lesions outside the mesial temporal lobes, mostly in the cortical and subcortical regions, and had no or minimal contrast enhancement. The epileptic foci demonstrated by EEG varied, including both temporal lobes and other lobes. Two patients had coexistent autoantibodies apart from GAD65 Abs, and 1 patient had a systemic autoimmune disorder in remission. However, none of the patients had a coexistent tumor. The prognoses of the patients were generally good with treatment of antiepileptic drug, steroids, IVIg, plasma change, and other immunosuppressive agents. This is the first case report of anti-GAD ELE with a tumor.

**Table 1:** Previously reported anti-glutamic acid decarboxylase 65 extralimbic encephalitis adult patients who had no limbic encephalitis.

Patient No.		#1	#2	#3	#4	#5
Literature		Kobayakawa Y et al 2010.^[21]^	Najjar S et al 2011.^[3]^	Najjar S et al 2011.^[3]^	Kojima G et al 2014.^[4]^	Triplett J et al 2018.^[22]^
Age		36	31	65	66	21
Sex		F	F	F	F	F
Clinical manifestation	Initial symptom	Psychiatric symptom (disturbance of perception)	GTCS	CPS	GTCS	EPC
	Seizure	Yes	Yes	Yes	Yes	Yes
	Seizure semiology (if exist)	Generalized	GTCS, partial	SPS, CPS	GTCS, SE, partial	EPC, TCS,
	Refractory seizure	Yes	Yes	Yes	Yes	Yes
	Accompanying symptom	Psychiatric symptom	No	Hand weakness, hemianopia, psychiatric symptom	No	Consciousness decrease, cognitive deficit, motor, and sensory disturbance
	Psychiatric or cognitive disturbance	Yes	No	Yes	No	Yes
CSF	WBC (/uL)	21 (all mononuclear)	Normal	Normal	25 (97% lymphocytes)	21 (all mononuclear)
	Protein (mg/dL)	25	Normal	Normal	Normal	53
	Oligoclonal band	+	-	+	NA	-
Brain MRI	T2/FLAIR MRI	Bilateral frontal, parietal and occipital lobes	Right anterior frontal and mesial occipitoparietal lobes; Right hemispheric white matter	Right temporal and frontal parietal operculum; Mesial occipital lobes	Right temporal lobe and left anterior superior frontal gyrus; Left temporal pole	Multiple bifrontal cortical and subcortical foci
	Contrast enhancement	-	+	+(minimally)	-	-
EEG		Epileptic activities with frequent sharp waves in bilateral frontal regions	Normal	Right temporal central slowing and epileptiform spikes	Periodic lateralizing epileptiform discharges from the left temporal area, and independent frequent epileptiform discharge from the right temporal area	Generalized background slowing, left frontal seizure, and multifocal independent periodic complexes
GAD65 Ab (serum, CSF)		12500 U/L, 476 U/L	115,000 IU/L	183,000 IU/L	<1:600 (weakly positive)	469U/L
Other autoantibodies		ANA, anti-SS-B	-	-	Anti-AChR, anti-striated muscle; TgAb, anti-TPO, RF, anti-CCP	-
Autoimmune disorder		No	No	No	MG (in remission 18m ago)	No
Malignancy		No	No	No	No	No
Treatment		AED, steroids, PE	AED, IVIg, steroids	AED, surgical resection, IVIg, steroids, azathioprine, MMF	AED, steroids, azathioprine, IVIg	AED, steroids, IVIg, PE, cyclophosphamide, rituximab, MMF
Outcome		Full recovery	Remission	Remission	Full recovery	Remission

Ab = antibody, AChR = acetylcholine receptor, AED = antiepileptic drug, ANA = antinuclear antibody, CCP = cyclic citrullinated peptide, CPS = complex partial seizure, CSF = cerebrospinal fluid, EEG = electroencephalogram, EPC = epilepsia partialis continua, FLAIR = fluid-attenuated inversion recovery, GAD65 = glutamic acid decarboxylase 65, GTCS = generalized tonic-clonic seizure, IVIg = intravenous immunoglobulin, MG = myasthenia gravis, MMF = mycophenolate mofetil, MRI = magnetic resonance imaging, NA = not available, PE = plasma exchange, RF = rheumatoid factor, SE = status epilepticus, SPS = simple partial seizure, TCS = tonic-clonic seizure, TgAb = thyroglobulin antibody, TPO = thyroid peroxidase, WBC = white blood cell.

GAD65-Ab is not a classic paraneoplastic Ab but had been reported in tumor including SCLC, breast cancer, thymoma, and testicular seminoma.^[19]^ A previous study found that patients with cancer and positive GAD Abs can develop neurological syndromes such as limbic and brainstem encephalitis, and opsoclonus–myoclonus syndrome.^[5]^ GAD65-epilepsies in the paraneoplastic context were very rare and usually related to LE and SPS.^[19,20]^ The possibility of an underlying tumor in GAD65-epilepsy increased if the patient was male, older than 60 years, presented with a classical PNS (e.g., SPS, cerebellar ataxia, and LE), and had other neuronal cell-surface antibodies such as γ-aminobutyric acid (B) receptor-Ab. The tumors most frequently were lung and thymic neoplasms, consistent with their propensities to induce autoimmune disorders.^[2]^ SCLC is a highly malignant neuroendocrine tumor and usually presented with paraneoplastic neurological autoimmunity. In a retrospective study of 116 SCLC patients, among those 71 patients who presented with PNS, only 4 had seizures, and 16 had positive GAD65 Abs in serum; among those 45 patients who did not present with PNS, GAD65 Ab was also the most common type of neural autoantibodies, with 14 having it.^[23]^ Therefore, GAD65 Ab is frequently positive in SCLC patients, and is likely associated with PNS in SCLC. In our patient, SCLC may relate to the production of GAD65 Abs and the pathogenesis of epilepsy. We infer that the pathogenic GAD65 Abs or GAD65-specific T-cells could be produced by tumor tissues in the lung, resulting from the immunity against tumors expressing GAD65 antigens. However, to confirm the pathogenic role of tumors in GAD65 autoimmunity, the pathological evidence that GAD65 is expressed in tumor tissues is necessary, and the exact mechanism of GAD65 Ab production and function may be required to be elucidated in further experimental studies.

## 4. Conclusion

We reported a case of a patient with anti-GAD65 ELE presented with isolated epilepsy, with a possible paraneoplastic etiology from SCLC. It suggests that anti-GAD65 encephalitis can be extralimbic, can present with isolated epilepsy, and can have an underlying tumor. What’s more, these rare scenarios can happen concurrently. This case expands the phenotypic spectrum of anti-GAD65 AE and contributes to the early identification and treatment of GAD65-epilepsy in the initial syndrome-based clinical evaluations. Additionally, it suggests the importance of early cancer screening in patients with cryptogenic epilepsy who had high titer anti-GAD56 Abs in their serums or CSF.

## Acknowledgments

We thank the patient for kindly agreeing to the reporting of this case.

## Author contributions

**Conceptualization:** Gao Chen.

**Data curation:** Si-Qi Xia, Hao-Nan Fan, Wu Xia.

**Investigation:** Wu Xia.

**Project administration:** Gao Chen.

**Supervision:** Gao Chen.

**Visualization:** Si-Qi Xia.

**Writing – original draft:** Si-Qi Xia.

**Writing – review & editing:** Si-Qi Xia, Hao-Nan Fan, Wu Xia.
